# Dysregulation of CircRNA_0001946 Contributes to the Proliferation and Metastasis of Colorectal Cancer Cells by Targeting MicroRNA-135a-5p

**DOI:** 10.3389/fgene.2020.00357

**Published:** 2020-05-08

**Authors:** Zhenwei Deng, Xiyao Li, Huaiming Wang, Yongyong Geng, Yongchang Cai, Yuxin Tang, Yijun Wang, Xueqiao Yu, Libo Li, Ruiping Li

**Affiliations:** ^1^Department of General Surgery, Dongguan People’s Hospital, Southern Medical University, Dongguan, China; ^2^Department of General Surgery, The First Hospital of China Medical University, Shenyang, China; ^3^Guangdong Provincial Key Laboratory of Colorectal and Pelvic Floor Diseases, Gastrointestinal Institute of Gastroenterology, The Sixth Affiliated Hospital of Sun Yat-sen University, Guangzhou, China; ^4^Department of Colorectal and Anal Surgery, Tumushuke People’s Hospital, Tumushuke, China; ^5^Department of Colorectal and Anal Surgery, Clinical Center of Intestinal and Colorectal Diseases of Hubei Province, Key Laboratory of Intestinal and Colorectal Diseases of Hubei Province, Zhongnan Hospital of Wuhan University, Wuhan, China

**Keywords:** circRNA_0001946, miR-135a-5p, colorectal cancer, proliferation, metastasis

## Abstract

This study was aimed to evaluate the potential function of circ-0001946 in the progression of colorectal cancer (CRC) and the related regulatory mechanism. First, the expression levels of circRNA_0001946 and microRNA-135a-5p (miR-135a-5p) in normal and CRC tissues were measured by quantitative real-time polymerase chain reaction (RT-qPCR). In addition, cell proliferation was assessed by the Cell Counting Kit-8 (CCK-8) assay, cell migration and invasion were evaluated by Transwell assays, and the cell cycle patterns were determined by flow cytometry. The relationship between the expression levels of circ_0001946 and miR-135a-5p was determined by dual-luciferase reporter assays. Our data showed that the expression of circ_0001946 was upregulated in CRC tissues, which was negatively correlated with tumor size, histologic grade, lymphatic metastasis, and TMN stage, and patients with circ_0001946 overexpression were more likely to have a poor prognosis. In addition, *in vitro* experiments showed that silencing circ_0001946 inhibited the epithelial–mesenchymal transition (EMT) pathway and markedly suppressed CRC cell growth, migration, and invasion. Furthermore, we discovered that the transfection of miR-135a-5p mimics could reverse the antitumor effects of circRNA_0001946 downregulation. To summarize, this study revealed that circRNA_0001946 might act as a tumor promoter by activating the miR-135a-5p/EMT axis and may be a promising treatment target for CRC.

## Introduction

Colorectal cancer (CRC) is the third most common malignant tumor and the second most common cause of cancer-related mortality worldwide (Torre et al., 2015; Bray et al., 2018). Radical gastrectomy combined with systemic chemotherapy is the current preferred treatment for patients with advanced CRC (Arnold et al., 2017). However, the survival time of CRC patients is not satisfactory (Garcia-Larsen et al., 2019) because many patients are initially diagnosed with advanced stage disease. Therefore, clarifying the underlying mechanism of CRC progression and identifying novel targets are of great importance.

Circular RNAs (circRNAs) are characterized by their unique closed loop structure (Wang et al., 2019b; Ye et al., 2019). In recent years, accumulating studies have demonstrated that circRNAs are involved in the progression of various cancers, including ovarian and liver cancer (Li et al., 2019; Wu et al., 2019), and emerging evidence has shown that circRNAs can sponge miRNAs and interfere with their biological function and thus participate in carcinogenesis (Meng et al., 2017; Cui et al., 2018; Verduci et al., 2019). For example, circ_0006168 can act as a tumor promoter by regulating the miR-100/mTOR axis (Shi et al., 2019). In addition, Wang et al. (2019c) showed that circ_0091570 sponged miR-1307 and suppressed carcinogenesis in hepatocellular cancer.

CircRNA_0001946, also known as CDR1as and CiRS-7, is a non-coding RNA that originates from chrX:139865339-139866824 and has been shown to be involved in the progression of various cancers, including CRC (Weng et al., 2017; Tang et al., 2017), lung adenocarcinoma (Yao et al., 2019), esophageal squamous cell cancer (Fan et al., 2019), and glioblastoma (Li and Diao, 2019). Li and Diao (2019) provided evidence that circ_0001946 plays an important role in the development of glioblastoma by regulating the miR-671-5p/CDR1 axis. However, the role and potential regulatory mechanisms of circRNA_0001946 in CRC are still not well understood.

Here, we first evaluated the expression of circRNA_0001946 in CRC tissues and its relationship with the clinicopathological characteristics of these CRC patients. We then performed *in vitro* experiments to evaluate the function of circRNA_0001946 in CRC progression. Based on the results of a bioinformatics analysis, we hypothesized that circRNA_0001946 could sponge miR-135a-5p and further enhance the tumorigenesis of CRC, and the relationship between circRNA_0001946 and miR-135a-5p was confirmed by dual-luciferase reporter assays. In summary, our data showed that circRNA_0001946 may be a promising therapeutic biomarker for CRC patients.

## Materials and Methods

### Patient Tissue Samples

A total of 64 paired CRC and normal tissues were collected from CRC patients who were admitted to Dongguan People’s Hospital of Southern Medical University for radical surgery between 2012 and 2014. All tissue samples were confirmed by experienced pathologists and were frozen and stored in a refrigerator of −80 degrees until use. All study patients provided written informed consent. This study was approved by the Medical Ethics Committee of Dongguan People’s Hospital of Southern Medical University.

### Cell Culture

Normal human colon epithelial cells (FHC) and five human CRC cancer cell lines (LoVo, SW480, Caco-2, SW620, and HT-29) were obtained from the American Type Culture Collection (ATCC; Shanghai, China). All cells were cultured in Dulbecco’s modified Eagle medium (DMEM; Gibco, Grand Island, NY, United States) containing 10% fetal bovine serum (FBS; Gibco) in a humidified incubator at 37°C and 5% carbon dioxide (CO_2_). Furthermore, in order to prevent the problem of mycoplasma contamination, all of the cell lines were treated with Mycoplasma Removal Agent (MP Biomedicals, United States) at the recommended concentration of 0.5 μg/ml.

### Cell Transfection

CircRNA_0001946-knockdown CRC cells were constructed by transfection with 5 μg/ml polybrene and specific lentiviruses [multiplicity of infection (MOI), 100]. Then, stably transfected, circRNA_0001946-knockdown cells (si-circRNA_0001946-1 and si-circRNA_0001946-2 cells) were obtained. These cells were then transfected with either a miR-135a-5p inhibitor or negative control (NC) sequence (miR-135a-5p NC) using Lipofectamine 3000 Transfection Reagent (Thermo Fisher Scientific, United States). The sequences of siRNAs are listed in Table 1.

**TABLE 1:** Sequences of oligomers and primers used in the present research.

Name	Sequence (5′–3′)
CircRNA_0001946 forward	CCA CGT CTT CCC AAC AAT CC
CircRNA_0001946 reverse	GAC CTG GAG GCC ATT GGA AG
miR-135a-5p forward	AGG GGT ATG GCT TTT TAT TCC
miR-135a-5p reverse	GTT GTG GTT GGT TGG TTT GT
GAPDH forward	CCT TCC GTG TCC CCA CT
GAPDH reverse	GCC TGC TTC ACC ACC TTC

### Quantitative Real-Time PCR Assay

Total RNA was extracted with TRIzol reagent (Takara, Dalian, China). Then, Prime Script RT Master Mix (Takara) was used to reverse transcribe 500 ng of the extracted RNA into cDNA according to the manufacturer’s protocol. Next, cDNA was amplified with the SYBR-Green PCR kit (Roche, United States). Finally, the 2^–ΔΔ*Ct*^ method was used to evaluate the expression levels of target genes. The complete sequences of the primers used are shown in Table 1.

### Cell Counting Kit-8 Assay

Treated cells (5 × 10^3^ cells/well) were collected and seeded into 96-well plates. After incubation for 24, 48, 72, and 96 h, 10% CCK-8 solution was added to each well, and the absorbance at 450 nm was determined with a microplate reader (Molecular Devices, Sunnyvale, CA, United States). Finally, cell viability was calculated based on the absorbance and compared.

### Transwell Assays

Transwell chambers (8 μm; Corning, NY, United States) with and without Matrigel (Corning) were used to assess cell migration and invasion, respectively. First, treated cells were collected and suspended in serum-free medium. Then, 100 μl of a cell suspension containing 4 × 10^4^ cells was placed in the top chamber, and 500 μl of DMEM containing 20% FBS was placed in the bottom chamber. After incubation, the cells trapped on the surface of Transwell chamber membrane were fixed with 4% paraformaldehyde and then stained with 0.1% crystal violet. Finally, these cells were imaged with a light microscope.

### Wound Healing Assay

Treated cells were collected and seeded in a six-well plate. Once the cells reached 90% confluence, an artificial wound was created in the center of the confluent cell monolayer with a 200 μl pipette tip. Then, the cells were incubated with DMEM containing 10% FBS for another 24 h, and would closure was evaluated under an inverted microscope (Olympus, Tokyo, Japan) at 100 × magnification.

### Flow Cytometric Analyses

Treated cells were collected, washed with PBS, and then fixed with 70% alcohol at 4°C overnight. Next, fixed cells were stained with a cell cycle detection kit (Keygen, Nanjing, China) and evaluated with a FACSCanto II flow cytometer (BD Biosciences) to determine the percentage of cells in various phases of the cell cycle.

### Dual-Luciferase Reporter Assays

The test (293T) cells were seeded in 24-well plates and incubated for 24 h. Then, the cells were co-transfected with either the pmirGLO-circRNA_0001946-WT or pmirGLO-circRNA_0001946-MUT plasmid along with either miR-135a-5p mimics or miR-135a-5p NC. After 48 h, relative luciferase activity was evaluated using the dual-luciferase reporter assay system (Promega, Madison, WI, United States).

### Western Blotting

Cells were treated with RIPA Lysis buffer (Beyotime, Shanghai, China) to generate crude protein extracts. Then, the protein concentration in the extracts was evaluated with the BCA Protein Assay Kit (Beyotime). Equal proteins were separated by 10% sodium dodecyl sulfate (SDS)-polyacrylamide gel electrophoresis (PAGE) and then transferred to polyvinylidene fluoride (PVDF). The blot was blocked with 10% bovine serum albumin (BSA) for 1 h, and then incubated with the primary antibody at 4°C overnight. The following primary antibodies were used: anti-E-cadherin (cat. no. ab15148), anti-N-cadherin (cat. no. ab245117), anti-vimentin (EPR3776) and anti-glyceraldehyde 3-phosphate dehydrogenase (GAPDH; cat. no. ab8245) (all from Abcam, Cambridge, MA, United States). The membranes were then incubated with an horseradish peroxidase (HRP)-conjugated anti-rabbit (cat. no. RABHRP1) or anti-mouse (cat. no. RABHRP2) secondary antibody (both from Sigma-Aldrich; Merck KGaA) for 1 h at room temperature. Finally, GeneSnap in the SynGene system was used to evaluate the protein bands.

### Statistical Analysis

Data are shown as the mean ± standard deviation (SD) and were analyzed with GraphPad Prism v7.0 software. The differences between groups were evaluated with Student’s *t*-test or the chi-square test. Moreover, comparisons among multiple groups were performed using one-way ANOVA. Differences with *P*-values less than 0.05 were considered significant.

## Results

### Upregulation of Circ_0001946 Predicts Poor Prognosis in Colorectal Cancer

**FIGURE 1:**
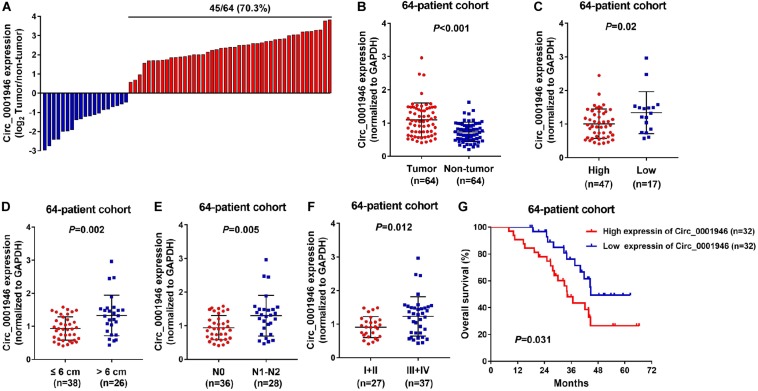
Upregulation of circ_0001946 predicts poor prognosis in colorectal cancer (CRC). **(A)** Circ_0001946 was significantly overexpressed in most (70.3%; 45/64) CRC samples. **(B)** Circ_0001946 expression was higher in CRC samples than in normal samples, as detected by quantitative real-time (RT-q)PCR. **(C)** The correlation between the expression of circ_0001946 and histologic grade. **(D)** The correlation between the expression of circ_0001946 and tumor size. **(E)** The correlation between the expression of circ_0001946 and lymphatic metastasis. **(F)** The correlation between the expression of circ_0001946 and TNM stage. **(G)** Higher circ_0001946 expression predicted worse overall survival by Kaplan–Meier analysis.

Figure 1A shows that circ_0001946 was overexpressed in 70.3% (45/64) of CRC specimens. Circ_0001946 was dramatically upregulated in the CRC tissue samples when compared to that in normal samples (Figure 1B; *P* < 0.001). CRC samples were placed into high and low expression groups based on the median expression of circ_0001946. The data also showed that circ_0001946 expression was highly related to tumor size (*P* = 0.000), histologic grade (*P* = 0.011), N stage (*P* = 0.002), TNM stage (*P* = 0.005), and carcinoembryonic antigen (CEA) level (*P* = 0.000; Table 2). CRC patients with circ_0001946 overexpression were more likely to have a low histologic grade (Figure 1C; *P* = 0.02), lymph node metastasis (Figure 1E; *P* = 0.005), advanced TNM stage (Figure 1F; *P* = 0.012), shorter overall survival time (Figure 1G; *P* = 0.0031), and larger tumors (Figure 1D; *P* = 0.002). These results showed that upregulation of circ_0001946 was closely associated with poor prognosis.

**TABLE 2:** Clinicopathological variables and the expression of circ_0001946 in colorectal cancer patients.

Clinicopathological variable	*N*	Circ_0001946		*P-*value
		High expression	Low expression	
**Age**				
≤60 years	34	14 (41.2%)	20 (58.8%)	0.133
>60 years	30	18 (60.0%)	12 (40.0%)	
**Gender**				
Male	40	23 (57.5%)	17 (41.5%)	0.121
Female	24	9 (37.5%)	15 (62.5%)	
**Tumor size**				
≤6 cm	38	12 (31.6%)	26 (68.4%)	0.000
>6 cm	26	20 (76.9%)	6 (23.1%)	
**Histologic grade**				
High	47	19 (40.4%)	28 (59.6%)	0.011
Low	17	13 (76.5%)	4 (23.5%)	
**N stage**				
N0	36	12 (33.3%)	24 (66.7%)	0.002
N1-N2	28	20 (71.4%)	8 (28.6%)	
**Tnm stage**				
I-II	27	8 (29.6 %)	19 (70.3%)	0.005
III-IV	37	24 (64.9%)	13 (35.1%)	
**Cea level**				
Normal (0–5 μg/L)	34	9 (26.5%)	25 (73.5%)	0.000
Elevated (>5 μg/L)	30	23 (76.7%)	7 (23.3%)	
**CA19-9 level**				
Normal (0–37 U/ml)	31	19 (61.3%)	12 (38.7%)	0.080
Elevated (>37 U/ml)	33	13 (39.4%)	20 (60.6%)	
**Total**	64	32 (50.0%)	32 (50.0%)	

*CEA, carcino-embryonic antigen; CA19-9, carbohydrate antigen 19-9.*

### Downregulation of Circ_0001946 Inhibits the Proliferation of Colorectal Cancer Cells and Arrests Colorectal Cancer Cells at G2/M Phase

**FIGURE 2:**
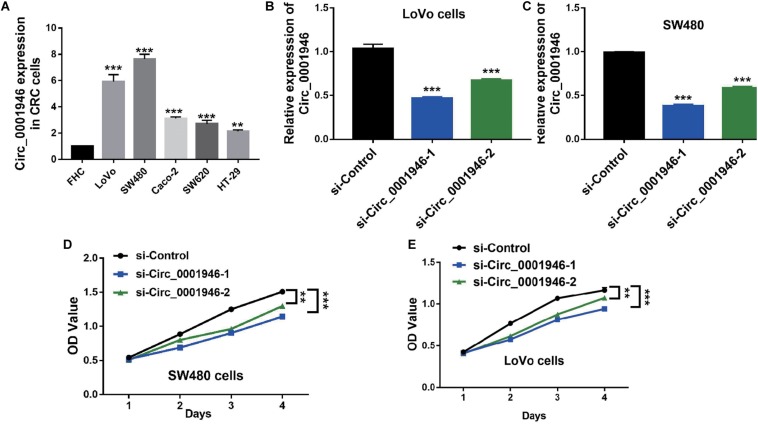
Downregulation of circ_0001946 inhibits the proliferation of colorectal cancer (CRC) cells. **(A)** The expression of circ_0001946 in CRC cell lines (LoVo, SW480, Caco-2, SW620, and HT-29) and immortalized normal intestinal cell line FHC. **(B)** Successful construction of circ_0001946-downregulated LoVo cells by a lentivirus-based method. **(C)** Successful construction of circ_0001946-downregulated SW480 cells by a lentivirus-based method. **(D)** The proliferation of circ_0001946-downregulated LoVo cells, as measured by the Cell Counting Kit-8 (CCK-8) assay. **(E)** The proliferation of circ_0001946-downregulated SW480 cells, as measured by the CCK-8 assay. All experiments were repeated at least three times. **P < 0.01, ***P < 0.001.

**FIGURE 3:**
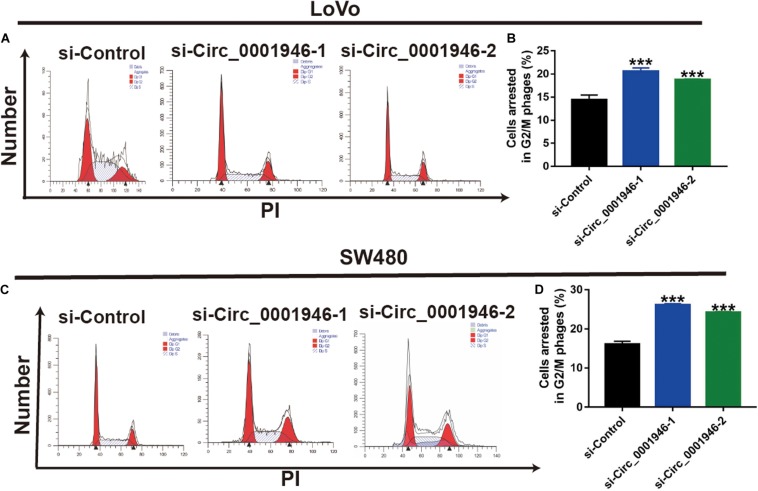
Downregulation of circ_0001946 arrests colorectal cancer (CRC) cells at G2/M phase. **(A)** Cell cycle images of LoVo cells transfected with circ_0001946-specific siRNAs. **(B)** Flow cytometry analysis of LoVo cells arrested at G2/M phase after transfection with circ_0001946-specific siRNAs. **(C)** Cell cycle images of SW480 cells transfected with circ_0001946-specific siRNAs. **(D)** Flow cytometry analysis of SW480 cells arrested at G2/M phase after transfection with circ_0001946-specific siRNAs. All experiments were repeated at least three times. ***P < 0.001.

The RT-qPCR results showed that, when compared with FHC cells, circ_0001946 was upregulated in the tested CRC cell lines (LoVo, SW480, Caco-2, SW620, and HT-29). LoVo and SW480 cells showed particularly high expression (Figure 2A). We knocked down circ_0001946 expression in these CRC cell lines by transfecting circ_0001946-specific siRNAs. The analysis showed that transfection of these siRNAs dramatically decreased circ_0001946 expression in LoVo and SW480 cells (referred to as the si-Circ_0001946-1 and si-Circ_0001946-2 groups) compared to the levels in negative control-transfected cells (si-Control). Si-Circ_0001946-1 cells showed higher inhibition efficiency (Figures 2B,C). These circ_0001946-knockdown cells were used in proliferation experiments, and the data indicated that knocking down circ_0001946 markedly suppressed the growth of LoVo and SW480 cells, as lower OD values were observed in the si-Circ_0001946 groups (Figures 2D,E). In addition, more si-Circ_0001946-transfected LoVo cells were arrested in G2/M phase than control-transfected LoVo cells (Figures 3A,B). Consistently, more circ_0001946-silenced SW480 cells were arrested at G2/M phase than control cells (Figures 3C,D).

### Downregulation of Circ_0001946 Inhibits Metastasis and Regulates the Epithelial–Mesenchymal Transition Pathway in Colorectal Cancer Cells

**FIGURE 4:**
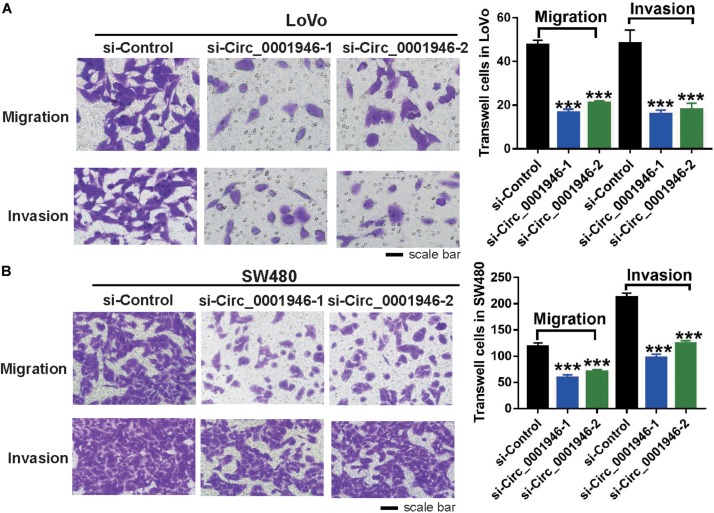
Downregulation of circ_0001946 inhibits the metastasis of colorectal cancer (CRC) cells. **(A)** Cell migration and invasion images of LoVo cells (left) and statistical analysis of migrated and invaded LoVo cells (right) after transfection with circ_0001946-specific siRNAs. **(B)** Cell migration and invasion images of SW480 cells (left) and statistical analysis of the migrated and invaded SW480 cells (right) after transfection with circ_0001946-specific siRNAs. All experiments were repeated at least three times. ***P < 0.001.

Analysis of the relationship between circ_0001946 and various clinicopathological parameters in patients with CRC showed that circ_0001946 upregulation was always accompanied by lymph node metastasis. Therefore, we speculated that circ_0001946 overexpression in CRC cells might be related to metastasis. Using a Transwell assay, we confirmed that depletion of circ_0001946 reduced the migration and invasion ability of CRC cells (Figure 4A). Consistently, we observed that compared to control cells, si-circ_0001946-transfected SW480 cells showed less migration and invasion (Figure 4B). Moreover, the results of the wound healing assay showed that the migratory distance was markedly reduced in LoVo and SW480 cells transfected with si-Circ_0001946 (Supplementary Figure S1).

**FIGURE 5:**
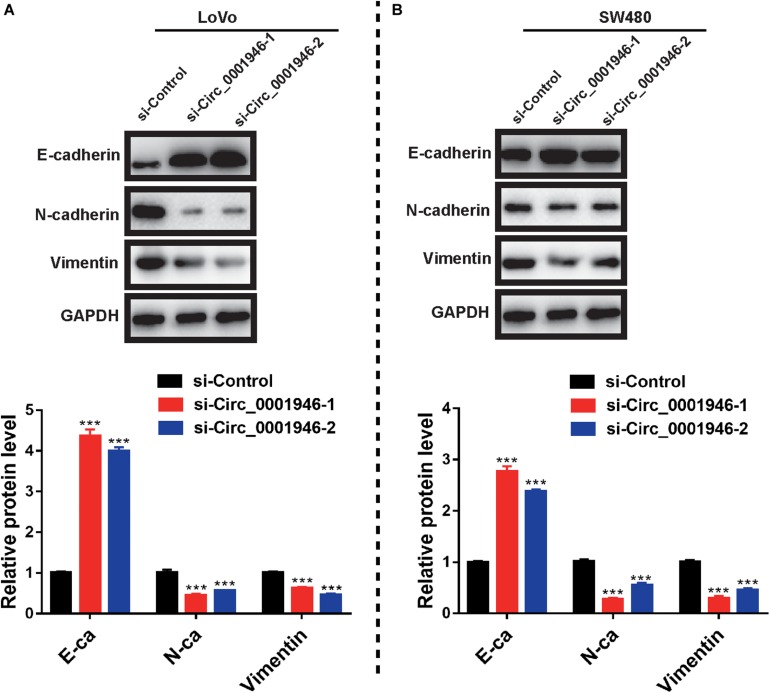
Downregulation of circ_0001946 inhibits the epithelial–mesenchymal transition (EMT) pathway in colorectal cancer (CRC) cells. **(A)** Expression of an epithelial marker (E-cadherin) and a mesenchymal marker (N-cadherin and vimentin) in LoVo cells (upper panel) and the representative gray value of LoVo cells (lower panel) after transfection with circ_0001946-specific siRNAs. **(B)** Expression of an epithelial marker (E-cadherin) and a mesenchymal marker (N-cadherin and vimentin) in SW480 cells (upper panel) and the representative gray value of SW480 cells (lower panel) after transfection with circ_0001946-specific siRNAs. All experiments were repeated at least three times. ***P < 0.001.

We assessed EMT-related markers in CRC cells and attempted to elucidate the molecular mechanism underlying circ_0001946-mediated regulation of cell proliferation and metastasis. Western blotting showed an increase in an epithelial-like marker (E-cadherin) and a decrease in a mesenchymal-like marker (N-cadherin and vimentin) following downregulation of circ_0001946 (Figures 5A,B). Taken together, we concluded that downregulation of circ_0001946 inhibited the proliferation and metastasis of CRC cells by regulating the EMT pathway.

### MiR-135a-5p Is a Downstream Target Gene of Circ_0001946 in Colorectal Cancer

**FIGURE 6:**
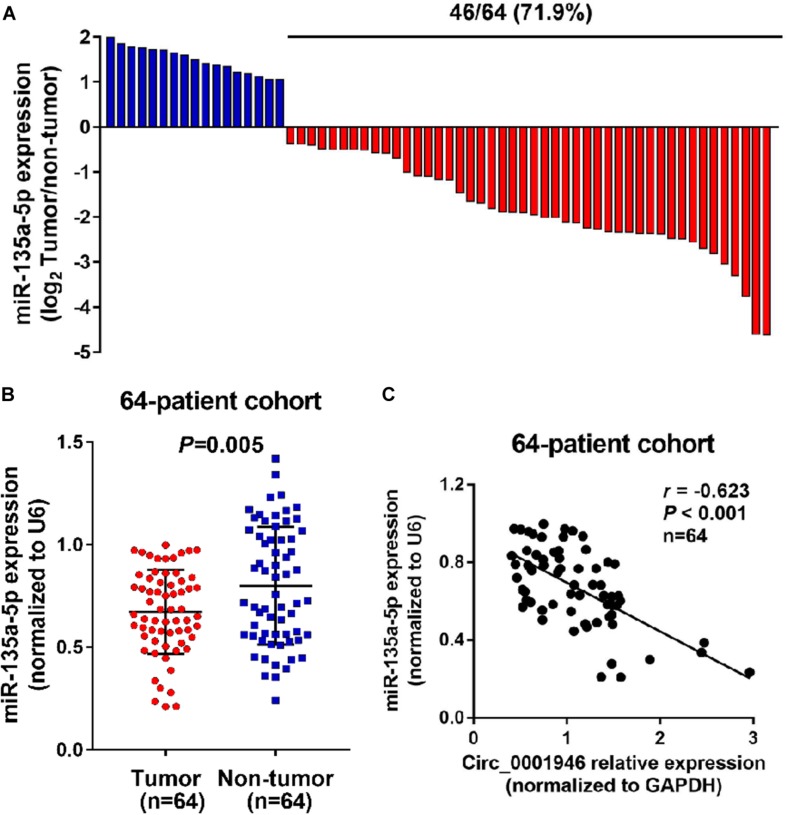
MiR-135a-5p is negatively related to circ_0001946 in colorectal cancer (CRC) samples. **(A)** MiR-135a-5p is significantly downregulated in 71.9% (46/64) of the tested CRC tissue samples. **(B)** MiR-135a-5p expression was lower in CRC samples than in normal samples, as determined by quantitative real-time (RT-q)PCR. **(C)** Negative relationship between circ_0001946 and miR-135a-5p in 64 paired samples from CRC patients.

**FIGURE 7:**
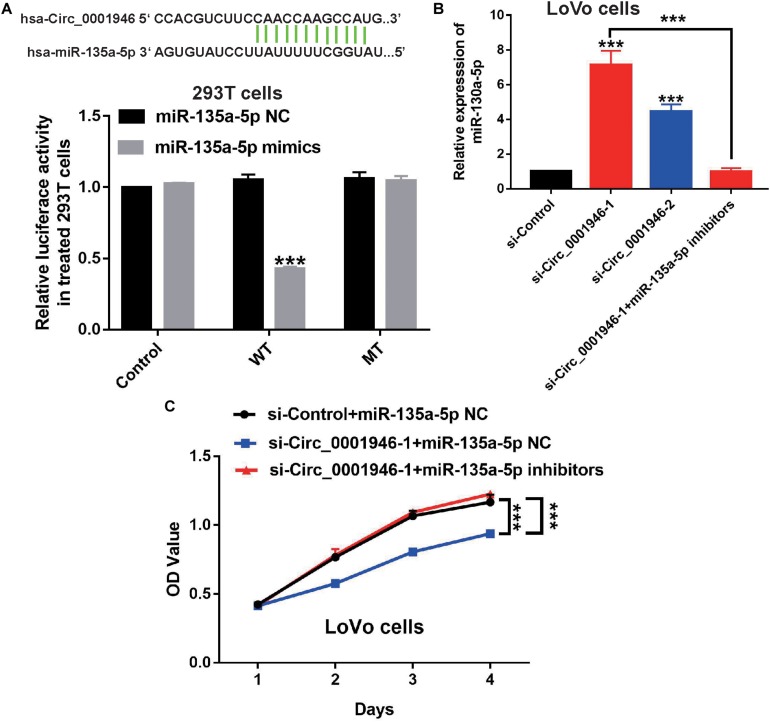
Downregulation of miR-135a-5p rescues the inhibition of colorectal cancer (CRC) cell proliferation induced by depletion of circ_0001946. **(A)** The predicted binding regions of circ_0001946 on miR-135a-5p (upper panel) and the relative luciferase activity in 293T cells after co-transfection with either pmirGLO-Circ_0001946-WT or pmirGLO-Circ_0001946-MUT, along with either miR-135a-5p specific mimics or negative control (NC). **(B)** The expression of miR-135a-5p in circ_0001946-downregulated LoVo cells and the successful downregulation of miR-135a-5p in LoVo cells after transfection with circ_0001946-specific siRNAs using miR-135a-5p inhibitors. **(C)** The growth of circ_0001946-downregulated LoVo cells, with or without depletion of miR-135a-5p, as detected by the Cell Counting Kit-8 (CCK-8) assay. All experiments were repeated at least three times. ***P < 0.001.

Based on the results of a bioinformatics analysis (Starbase.2), we predicted that miR-135a-5p is a downstream target of circ_0001946. Therefore, we evaluated the expression of miR-135a-5p in CRC samples. The results showed low miR-135a-5p expression in 71.9% (46/64) of CRC specimens (Figure 6A). MiR-135a-5p expression was dramatically downregulated in CRC tumor samples compared to the levels in normal tissue samples (Figure 6B), and miR-135a-5p expression was negatively associated with circ_0001946 expression (Figure 6C). To further explore this relationship, we predicted their potential binding sites, which are shown in Figure 7A. Then, their relationship was evaluated in a luciferase reporter assay, and wild-type cells treated with miR-135a-5p mimics displayed lower luciferase activity (Figure 7A). We also assessed miR-135a-5p levels in LoVo cells. Interestingly, si-Circ_0001946-transfected cells showed higher miR-135a-5p expression than si-control-transfected cells, and cells in the si-Circ_0001946-1 group showed the highest miR-135a-5p expression (Figure 7B). These results show that miR-135a-5p may be a direct target gene of circ_0001946 in CRC.

### Downregulation of MiR-135a-5p Rescued the Decrease in Colorectal Cancer Cell Proliferation and Metastasis Induced by Depletion of Circ_0001946

**FIGURE 8:**
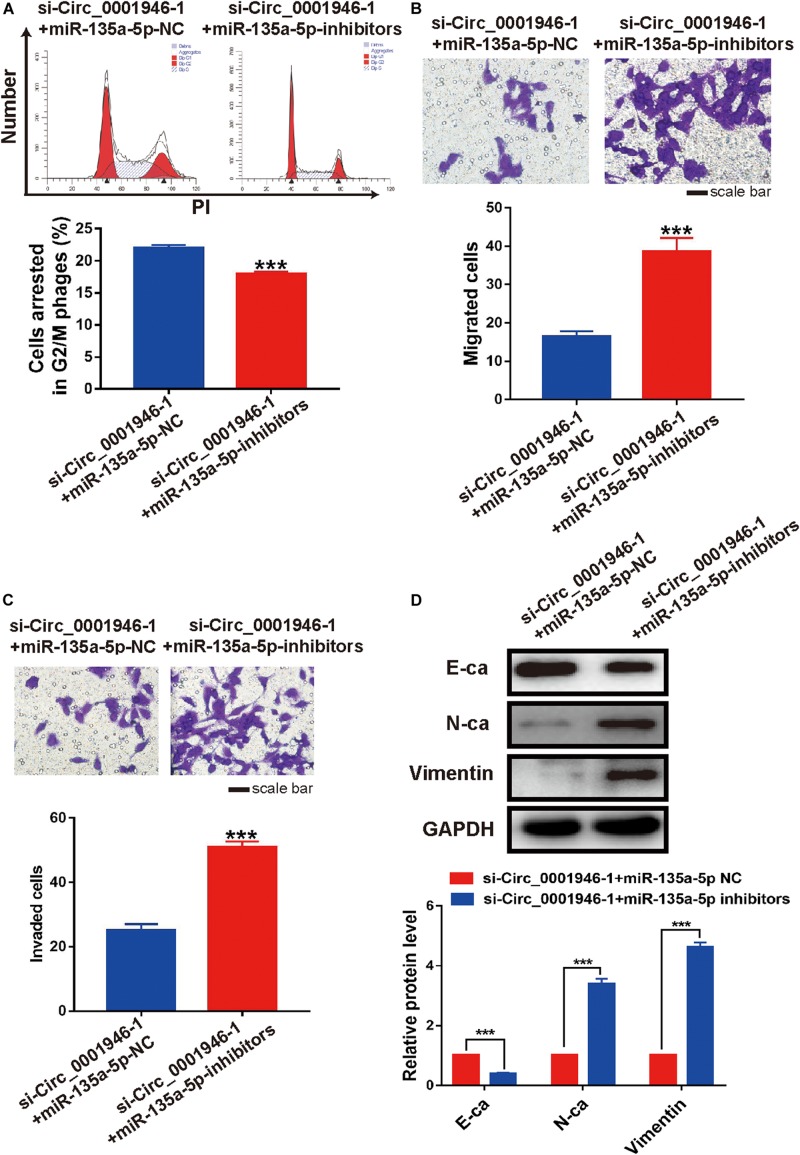
Downregulation of miR-135a-5p rescues the cell cycle, metastasis, and epithelial–mesenchymal transition (EMT) of colorectal cancer (CRC) cells induced by depletion of circ_0001946. **(A)** Cell cycle analysis of circ_0001946-downregulated LoVo cells after transfection of miR-135a-5p negative control (NC) or miR-135a-5p inhibitors. **(B)** Cell migration of circ_0001946-downregulated LoVo cells after transfection with miR-135a-5p NC or miR-135a-5p inhibitors. **(C)** Cell invasion of circ_0001946-downregulated LoVo cells after transfection with miR-135a-5p NC or miR-135a-5p inhibitors. **(D)** The expression of EMT-related proteins in circ_0001946-downregulated LoVo cells transfected with miR-135a-5p NC or miR-135a-5p inhibitors. All experiments were repeated at least three times. ***P < 0.001.

We decreased miR-135a-5p expression in si-Circ_0001946-1-transfected LoVo cells using miR-135a-5p-specific inhibitors (Figure 7B). The results of a CCK-8 assay showed that the miR-135a-5p inhibitors rescued the depleted cell proliferation ability in circ_0001946-knockdown LoVo cells (Figure 7C). Furthermore, downregulation of miR-135a-5p reduced cell cycle progression compared with that in miR-135a-5p NC cells (Figure 8A). Transwell assays showed that downregulation of miR-135a-5p also reduced cell migration and invasion (Figures 8B,C). Consistently, the results of the wound healing assays also showed that downregulation of miR-135a-5p reduced cell migration (Supplementary Figure S2). We also detected EMT-related markers in CRC cells after depletion of miR-135a-5p. Western blotting showed a decrease in the epithelial-like marker E-cadherin and an increase in the mesenchymal-like marker N-cadherin and vimentin (Figure 8D). Therefore, we concluded that downregulation of circ_0001946 suppressed cell proliferation, migration, and invasion in CRC by sponging miRNA-135a-5p.

## Discussion

Although CRC is a common malignant tumor worldwide (Bray et al., 2018), the mechanisms involved in the tumorigenesis of CRC are still not well understood (Torre et al., 2015). In the past few years, numerous studies have shown that dysfunction of circRNAs is a key regulatory pathway in the development of various cancers. For instance, a previous study showed that circRNA_100269 acts as a suppressor in gastric cancer (Zhang et al., 2017). Therefore, identifying novel circRNAs related to the development of CRC and elucidating the potential regulatory mechanisms are of great importance.

The results of these studies showed that circ_0001946 expression was obviously upregulated in CRC tissues relative to the levels in normal tissues. Our data showed that circ_0001946 expression levels were negatively associated with tumor size, histologic grade, lymphatic metastasis, and TMN stage and that circ_0001946 overexpression was often associated with poor prognosis. A previous study showed that circRNA_0001946 increased cell growth and metastases in hepatocellular cancer. Similarly, the results of this study also indicated that silencing of circRNA_0001946 arrested CRC cells at G2/M phase and promoted cell proliferation. Furthermore, the Transwell assay results consistently revealed that circRNA_0001946 downregulation suppressed CRC cell migration and invasion. However, the role of circRNA_0001946 in the carcinogenesis and development of CRC is still not clear.

The EMT is a pivotal biological process in which cells lose their epithelioid characteristics and gain mesenchymal characteristics that are closely associated with the promotion of malignancy (Savagner, 2015; Brabletz et al., 2018; Li and Shen, 2019). Numerous recent studies demonstrated that the EMT signaling pathway could be triggered by circRNA dysfunction (Zhou et al., 2018). For example, a previous study demonstrated that downregulation of circ_0067934 could suppress the EMT signaling pathway, inhibit cell proliferation, and promote apoptosis (Wang et al., 2019a). In addition, Wang et al. (2018) demonstrated that circRNA_0008305 inhibited transforming growth factor (TGF)-β-induced EMT, which contributed to the inhibition of cell metastasis in non-small-cell lung cancer. The data in our present study showed that silencing of circ_0001946 could upregulate the epithelial-like marker E-cadherin and downregulate the mesenchymal-like marker N-cadherin and vimentin, suggesting that the carcinogenic effects of circ_0001946 might be mediated by regulation of the EMT signaling pathway.

Several studies have demonstrated that circRNAs directly inhibit the function of miRNAs through cavernous mechanism, resulting in tumor suppression or cancer promotion effects. For example, circ_100290 functions as a competing endogenous RNA (ceRNA) for miR-378a, which promotes oral squamous cell carcinoma progression (Chen et al., 2019). Other studies indicated that circRNA_0020123 participates in the development of lung cancer by interfering with the miR-488-3p/ADAM9 axis. Therefore, we searched for downstream target genes of circRNA_0001946 in a bioinformatics analysis and identified miR-135a-5p as a potential target. The relationship between miR-135a-5p and circRNA_0001946 was confirmed in a dual-luciferase reporter assay.

Recently, increasing attention has been paid to elucidating the biological functions of miRNAs, and miR-135a-5p has been shown to be involved in the progression of various cancers, including lung adenocarcinoma, ovarian cancer, and thyroid carcinoma, among others. For example, Wang et al. (2017) revealed that serum miR-135a-5p was upregulated in CRC patients, suggesting that serum miR-135a-5p may prove to be an important biomarker for auxiliary diagnosis of CRC. In addition, another study indicated that circ_0001946 could promote cell growth in lung adenocarcinoma by regulating miR-135a-5p/SIRT1 axis and activating Wnt/β-catenin signaling pathway (Yao et al., 2019). Similarly, the results of the present study showed that miR-135a-5p expression in tumor tissues was higher than that in normal tissues and that miR-135a-5p expression was negatively related to the expression of circRNA_0001946. Moreover, our data indicated that an miR-135a-5p mimic could reverse the inhibition of tumor progression induced by silencing of circRNA_0001946. However, there are still several limitations in this article. Firstly, the downstream target gene of miR-135a-5p needs further research to confirm. Secondly, we failed to verify whether downregulation of circRNA_0001946 could also perform antitumor effects *in vivo*. Taken together, these results show that circRNA_0001946 was involved in the progression of CRC by sponging miR-135a-5p.

## Conclusion

In conclusion, our data showed that silencing of circRNA_0001946 directly regulated the function of miR-135a-5p, resulting in EMT signaling pathway interference, followed by arrest at G2/M phase and suppression of cell proliferation and metastasis in CRC. However, there are some limitations in this study. First, the molecules downstream of the circRNA_0001946/miR-135a-5p axis are unknown. Second, *in vivo* experiments are needed to confirm these results. Overall, our findings indicate that circRNA_0001946 acts as an oncogene in CRC and is a promising target for the treatment of CRC.

## Data Availability Statement

The datasets used during the present study are available from the corresponding author upon reasonable request.

## Ethics Statement

All study patients provided written informed consent. This study was approved by the medical ethics committee of Dongguan People’s Hospital of Southern Medical University.

## Author Contributions

ZD, XL, and RL conceived and designed the study. HW, YG, XY, and LL performed the experiments. YC, YT, and YW wrote the manuscript. All co-authors have read the manuscript and agreed with its content. This manuscript was revised by all authors.

## Conflict of Interest

The authors declare that the research was conducted in the absence of any commercial or financial relationships that could be construed as a potential conflict of interest.

## Supplementary Material

FIGURE S1 The Results of the wound healing assay and the statistics analysis of LoVo cells **(A)** and SW480 cells **(B)**, after transfected with Circ_0001946-specific siRNAs. ^∗∗^*P* < 0.01, ^∗∗∗^*P* < 0.001.

FIGURE S2 The Results of the wound healing assay and the statistics analysis of circ_0001946-downregulated LoVo cells **(A)** and circ_0001946-downregulated SW480 cells **(B)**, with the transfection of miR-135a-5p NC or miR-135a-5p inhibitors. ^∗∗^*P* < 0.01, ^∗∗∗^*P* < 0.001.
