# Supplementary material for: Dysregulation of CircRNA_0001946 Contributes to the Proliferation and Metastasis of Colorectal Cancer Cells by Targeting MicroRNA-135a-5p
Source: Front Genet. 2020 May 8;11:357. doi: 10.3389/fgene.2020.00357 (PMC7232565; doi:10.3389/fgene.2020.00357)
Supplement: FIGURE S1 — The Results of the wound healing assay and the statistics analysis of LoVo cells (A) and SW480 cells (B), after transfected with Circ_0001946-specific siRNAs. ∗∗P < 0.01, ∗∗∗P < 0.001. [file Data_Sheet_1.doc]

***Supplementary files***

***
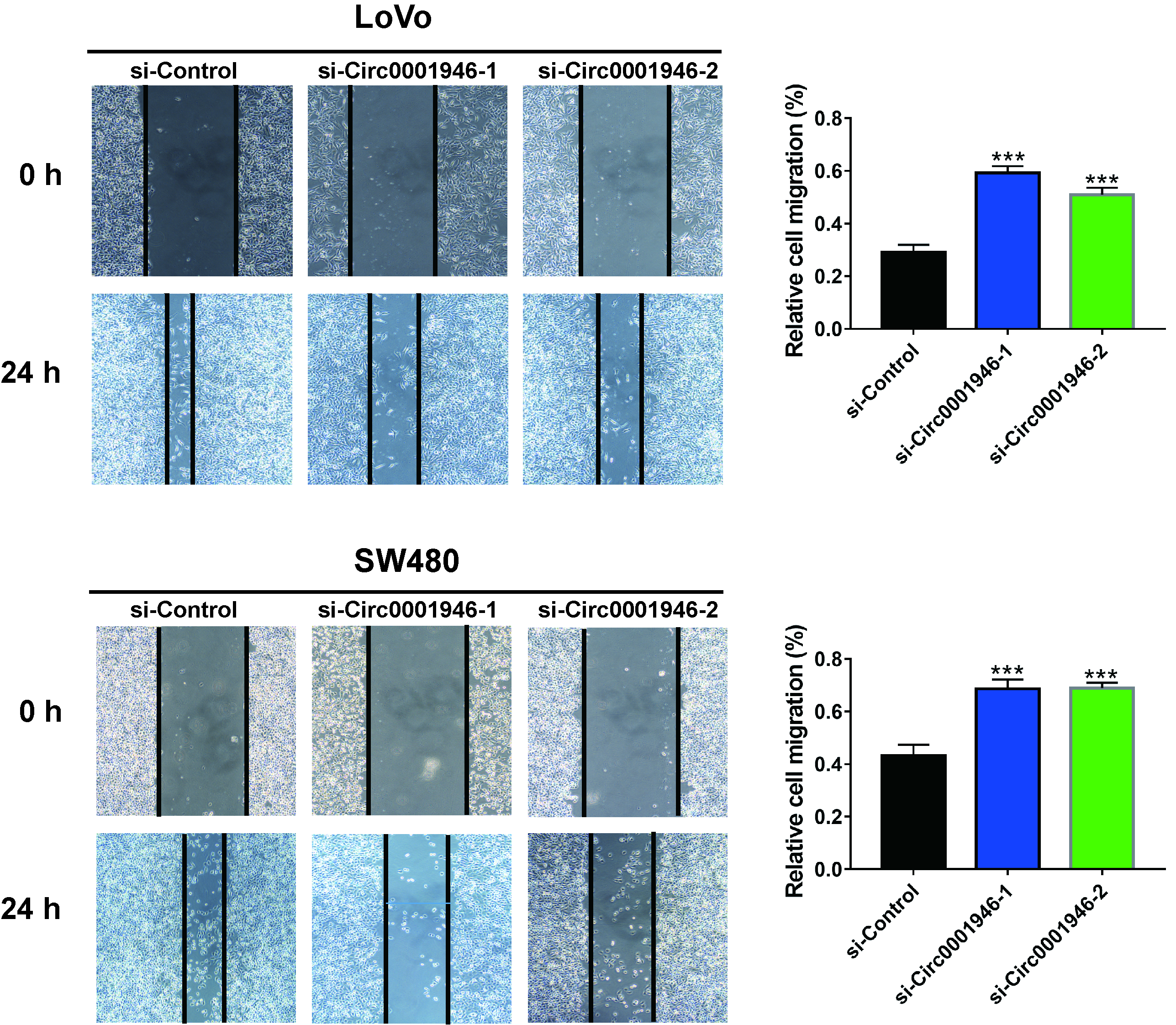
***

***Figure S1.*** ***T******he Results of the wound healing assay and the statistics analysis of LoVo cells (*A*) and SW480 cells (*B*), after transfected with Circ_0001946-specific siRNAs. **P<0.01, ***P<0.001***

***
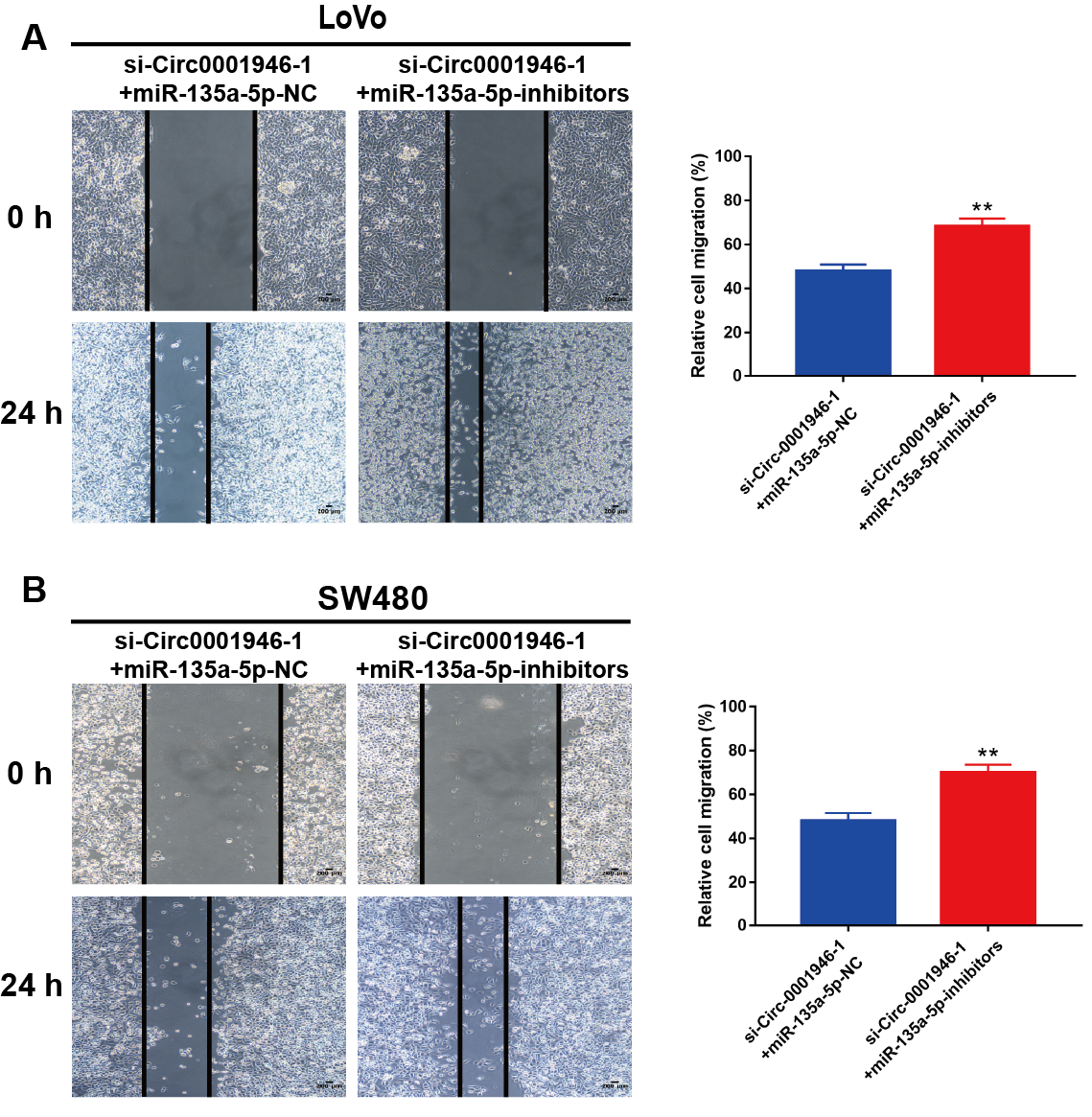
***

***Figure S2. The Results of the wound healing assay and the statistics analysis of circ_0001946-downregulated LoVo cells (*A*) and circ_0001946-downregulated SW480 cells (*B*), with the transfection of miR-135a-5p NC or miR-135a-5p inhibitors. **P<0.01, ***P<0.001***
